# S-Equol mitigates motivational deficits and dysregulation associated with HIV-1

**DOI:** 10.1038/s41598-021-91240-0

**Published:** 2021-06-04

**Authors:** Kristen A. McLaurin, Sarah J. Bertrand, Jessica M. Illenberger, Steven B. Harrod, Charles F. Mactutus, Rosemarie M. Booze

**Affiliations:** Program in Behavioral Neuroscience, Department of Psychology, Barnwell College, University of South Carolina, 1512 Pendleton Street, Columbia, SC 29208 USA

**Keywords:** Motivation, HIV infections, Spine structure

## Abstract

Motivational deficits (e.g., apathy) and dysregulation (e.g., addiction) in HIV-1 seropositive individuals, despite treatment with combination antiretroviral therapy, necessitates the development of innovative adjunctive therapeutics. S-Equol (SE), a selective estrogen receptor β agonist, has been implicated as a neuroprotective and/or neurorestorative therapeutic for HIV-1 associated neurocognitive disorders (HAND); its therapeutic utility for motivational alterations, however, has yet to be systematically evaluated. Thus, HIV-1 transgenic (Tg) and control animals were treated with either a daily oral dose of SE (0.2 mg) or vehicle and assessed in a series of tasks to evaluate goal-directed and drug-seeking behavior. First, at the genotypic level, motivational deficits in HIV-1 Tg rats treated with vehicle were characterized by a *diminished* reinforcing efficacy of, and sensitivity to, sucrose. Motivational dysregulation was evidenced by *enhanced* drug-seeking for cocaine relative to control animals treated with vehicle. Second, treatment with SE ameliorated both motivational deficits and dysregulation in HIV-1 Tg rats. Following a history of cocaine self-administration, HIV-1 Tg animals treated with vehicle exhibited lower levels of dendritic branching and a shift towards longer dendritic spines with decreased head diameter. Treatment with SE, however, led to long-term enhancements in dendritic spine morphology in HIV-1 Tg animals supporting a potential underlying basis by which SE exerts its therapeutic effects. Taken together, SE restored motivated behavior in the HIV-1 Tg rat, expanding the potential clinical utility of SE to include both neurocognitive and affective alterations.

## Introduction

Motivation, a multi-faceted construct, is an adaptational system that utilizes internal and external conditions to regulate behavior. Mechanistically, motivation is regulated by the fronto-striatal circuit (for review^1^), which includes the ventral tegmental area (VTA), nucleus accumbens (NAc) and prefrontal cortex (PFC). Disruption of the fronto-striatal circuit manifests in motivational alterations (for review^2^), which fall along a continuum and can be broadly categorized as motivational deficits (e.g., apathy), dysregulation (e.g., addiction), and/or excess (e.g., bipolar disorder). Etiologies, including human immunodeficiency virus type 1 (HIV-1; for review^3^), that target the neural substrates of the fronto-striatal circuit are commonly associated with prominent motivational alterations.

Indeed, HIV-1 seropositive individuals exhibit an increased neurobehavioral burden, evidenced by a greater prevalence of apathy^4–6^, addiction^7^, and bipolar disorder^7^, relative to their seronegative counterparts. Motivational alterations are associated with profound functional consequences, including greater impairment in activities of daily living^4,8^, an increased number of cognitive complaints^4,8^ and neurocognitive impairments^9^, decreased medication adherence^9–11^, and decreased quality of life^5,12^. Thus, there remains a critical need to develop innovative adjunctive therapeutics to mitigate the prominent motivational alterations in HIV-1 seropositive individuals.

Gamma-aminobutyric acid (GABA) medium spiny neurons (MSNs) of the NAc, a key component of the fronto-striatal circuit, play a central role in regulating motivational behaviors (e.g.,^13^). Accounting for approximately 95% of the cells within the region^14^, MSNs are characterized by a centrifugal morphology and high densities of dendritic spines^15^. The morphological parameters of dendritic spines are tightly coupled with synaptic function (e.g.,^16–19^), affording a unique tool to infer the function of spine synapses (for review^20^). Indeed, long-term HIV-1 viral protein exposure induces a prominent distributional shift in the morphology of dendritic spines, characterized by decreased dendritic spine volume^21,22^ and increased dendritic spine length^22^; morphological parameters which support synaptic dysfunction. Alterations in the dendritic branching complexity^21,22^ and neuronal excitability^23^ of MSNs have also been observed following chronic HIV-1 viral protein exposure. Most critically, MSNs have been implicated as a key structural locus for the actions of HIV-1 viral proteins on goal-directed behaviors^22^ supporting a key target for the development of innovative therapeutics.

Equol, a phytoestrogen that is structurally similar to 17β-estradiol^24^, may serve as a novel therapeutic to target the prominent synaptic dysfunction observed in MSNs following chronic HIV-1 viral protein exposure. Following the ingestion of the soy derived phytoestrogen daidzein, Equol is produced by the gut microbiota^25^. S-Equol (SE), the only enantiomer produced by humans^26^, penetrates the central nervous system via the blood–brain barrier and exhibits selective affinity for estrogen receptor β (ERβ;^26,27^). ERβ is widely distributed in the fronto-striatal circuit, including in the PFC and VTA^28^. Critically, 17β-estradiol alters the structure of MSNs in the NAc^29,30^. With regards to HIV-1 viral proteins, pretreatment with SE precludes synapse loss resulting from exposure to Tat^27^; research which corroborates studies demonstrating the utility of daidzein, a precursor to SE^25^, to both protect and restore synaptodendritic damage due to HIV-1 viral proteins^31^. Furthermore, SE has been implicated as an efficacious therapeutic for the neurocognitive impairments, collectively termed HIV-1 associated neurocognitive disorders (HAND), associated with the disease^32–34^ heralding an investigation of its utility for motivational alterations associated with HIV-1.

Thus, the goals of the present study were twofold. First, to systematically evaluate the therapeutic efficacy of SE for motivational alterations in the HIV-1 Tg rat. Goal-directed and drug-seeking behavior, indices of apathy and addiction, respectively, were investigated using operant conditioning. Second, to evaluate a potential underlying basis for the therapeutic effects of SE for motivational alterations. The morphology of MSNs of the NAc, and associated dendritic spines, was examined using a ballistic labeling technique after the completion of behavioral assessments. Given that pharmacological treatments for apathy^35^ and/or addiction^36^ are currently limited, the development of a novel therapeutic for motivational alterations associated with HIV-1 has the potential for broad clinical significance.

## Methods

### Animals

The efficacy of SE as an innovative therapeutic for motivational alterations associated with chronic HIV-1 viral protein exposure were evaluated in ovariectomized (OVX) female Fisher (F344/NHsd; Harlan Laboratories, Inc., Indianapolis, IN) HIV-1 Tg (*n* = 21) and control (*n* = 21) rats. The HIV-1 Tg rat, originally reported by Reid et al.^37^, expresses 7 of the 9 HIV-1 viral proteins (i.e., *env*, *tat, rev, vif, vpr, vpu, nef*) constitutively throughout development^38,39^. Although the HIV-1 Tg rat is rendered non-infectious by the deletion of *gag* and *pol*, the biological system expresses viral proteins relevant to central nervous system damage (e.g., *gp120*^40^, *tat*^31,41^). The HIV-1 Tg rat exhibits relatively good health throughout the functional lifespan, evidenced by similar growth rates relative to control animals^21,38^ and intact sensory and motor system function^38,42^.

Unrelated animals were requested to prevent the violation of the independent observation assumption inherent in many traditional statistical techniques. Furthermore, given the potential for sporadic transgene insertion elsewhere in the rat genome, age-matched F344/NHsd controls (rather than littermates) were purchased from Harlan Laboratories to assure the most developmentally appropriate and genetically stable baseline.

Animals were delivered to the animal vivarium, after being OVX at Harlan Laboratories, between six and eight months of age. SE, the therapeutic of interest, is a nonsteroidal estrogen that exhibits a selective affinity for ERβ^26,27^. Therefore, to preclude any potential confounding effects of endogenous hormones, female animals were OVX and fed a minimal phytoestrogen diet (≤ 20 ppm; Teklad 2020X Global Extruded Rodent Diet (Soy Protein-Free)). Animals had ad libitum access to rodent food and water, unless otherwise specified.

Animals were maintained in AAALAC-accredited facilities according to guidelines established by the National Institutes of Health and the ARRIVE guidelines. The animal colony was maintained at 21 ± 2 °C, 50 ± 10% relative humidity and a 12-h light:12-h dark cycle with lights on at 0700 h (EST). The Institutional Animal Care and Use Committee (IACUC) at the University of South Carolina (Federal Assurance #D16-00028) approved all experimental procedures. The study was carried out following all the relevant National Institutes of Health guidelines.

### Apparatus

Assessments of goal-directed and drug-seeking behavior were conducted in operant chambers (ENV-008; Med-Associates, St. Albans, VT, USA) located within sound-attenuating enclosures and controlled by Med-PC computer interface software. The front panel of the operant chamber contained a magazine that allowed a recessed 0.01 cc dipper cup (ENV-202C) to deliver a solution through a 5 cm × 5 cm opening (ENV 202M-S), two retractable “active” metal levers (i.e., responding resulted in reinforcement; ENV-112BM), and two white cue lights (28 V). Head entries into the magazine were detected using an infrared sensor (ENV 254-CB). To control for side bias, an “active” lever retracted after five consecutive presses on a single lever. The rear panel of the operant chamber had one non-retractable “inactive” lever, whereby responding was recorded, but not reinforced, and a house light (28 V).

During the assessment of drug-seeking behavior, intravenous cocaine infusions were delivered through a water-tight swivel (Instech 375/22ps 22GA; Instech Laboratories, Inc., Plymouth Meeting, PA), which was connected to the backmount of the animal using Tygon tubing (ID, 0.020 IN; OD, 0.060 IN) enclosed by a stainless steel tether (Camcaths, Cambridgeshire, Great Britain), using a syringe pump (PHM-100). A Med-PC computer program was utilized to calculate pump infusion times based on an animal’s daily bodyweight.

### Drugs

Cocaine hydrochloride (Sigma-Aldrich Pharmaceuticals, St. Louis, MO) was weighed as a salt and was dissolved in physiological saline (0.9%; Hospira, Inc., Lake Forest, IL). To preclude any significant hydrolysis of cocaine^43^, solutions were prepared immediately prior to the start of each testing session for every animal. Sucrose solutions were prepared at the beginning of each testing day.

After obtaining SE from Cayman Chemical Company (Ann Arbor, MI, USA), it was incorporated into 100 mg sucrose pellets (0.05 mg SE per sucrose pellet) by Bio-Serv (Flemington, NJ). Sucrose pellets (100 mg) were purchased from Bio-Serv for the vehicle group.

### Experimental timeline

**Figure 1:**
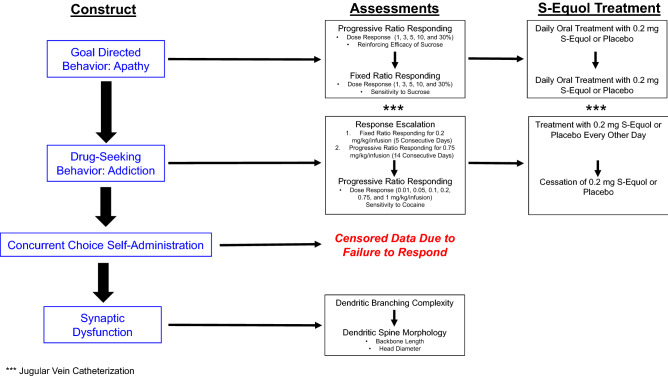
Experimental design schematic.

The experimental timeline for SE treatment, evaluation of goal-directed and drug-seeking behavior as well as neuroanatomical assessments, is illustrated in Fig. 1.

### Treatment

Animals were randomly assigned to receive either 0.2 mg SE (Control: *n* = 11; HIV-1 Tg: *n* = 11) or vehicle (Control: *n* = 10; HIV-1 Tg: *n* = 10). Between approximately 7 and 9 months of age, and one week prior to the start of operant training, HIV-1 Tg and control animals began receiving a daily treatment of either SE or vehicle. Given that each sucrose pellet contained 0.05 mg SE, animals receiving SE treatment received four pellets per day. Animals receiving vehicle treatment received four sucrose pellets per day. Daily treatment continued until jugular vein catheterization. Following jugular vein catheterization, animals were not treated for one week. When treatment resumed, it was given every other day until the end of the 14-day cocaine self-administration progressive ratio (PR) task. No treatment occurred during the cocaine self-administration PR dose–response task or concurrent choice self-administration. In total, HIV-1 Tg and control animals were treated for 84 days.

The 0.2 mg dose of SE was selected for two complementary reasons. First, using a dose–response experiment design, 0.2 mg SE was established as the most efficacious dose for the alleviation of sustained attention deficits in the HIV-1 Tg rat^32^. Second, the dose selected yielded a daily amount of 0.25–1.0 mg/kg SE (i.e., equivalent to a 2.5–10 mg dose in a 60 kg human); a dose well below the daily isoflavone intake of most elderly Japanese (i.e., 30–50 mg;^44^).

### Preliminary training

Previously established research protocols were used to conduct dipper training and autoshaping^45,46^. During dipper training, animals were trained to approach the magazine and drink a 5% sucrose solution (w/v) from the dipper receptacle. Subsequently, during autoshaping, animals learned to lever press for the 5% sucrose solution (w/v) using a fixed-ratio (FR) 1 schedule of reinforcement. Water restriction (12–15 h prior to assessment) was implemented throughout preliminary training. Animals had ad libitum access to water for 9–12 h after the completion of testing.

To successfully acquire autoshaping, animals were required to achieve at least 60 reinforcers for 3 consecutive days. Ad libitum access to water was reinstated following the successful completion of preliminary training. Water was available ad libitum for all subsequent assessments.

### Goal-directed behavior: apathy

Recognized as a multidimensional syndrome resulting in diminished motivation^8^, apathy is characterized by a quantitative reduction in voluntary and purposeful (goal-directed) behaviors^47^.

#### Progressive-ratio responding: dose response

Under a PR schedule of reinforcement, the response requirements are increased immediately following the delivery of a reinforcer^48^ affording a method to evaluate reinforcing efficacy^49^.

Following one maintenance session (i.e., 5% sucrose on an FR-1 schedule of reinforcement), animals were assessed using a PR schedule of reinforcement (Maximum Session Length: 120 Minutes). A dose–response experimental design was utilized, whereby the reinforcer was one of five sucrose concentrations (1, 3, 5, 10, and 30% w/v), presented using a Latin-Square experimental design, on test days, which occurred every other day. A maintenance session also occurred on intervening non-test days.

During PR tests, the ratio requirement was completed by responding across the two “active” levers. After successfully meeting the ratio requirement, the active levers were retracted and animals had 4 s of access to sucrose. The ratio requirement (rounded to the nearest integer) increased progressively according to the following exponential function: [5e^(reinforcer number × 0.2)^] − 5^49^.

#### Fixed-ratio responding: dose response

Under an FR schedule of reinforcement, the animal receives a reinforcer following a set number of responses (e.g., 1). Varying the unit-dose of sucrose changes responding, affording an opportunity to evaluate sensitivity to sucrose.

Animals responded for the same dose–response sucrose concentrations (i.e., 1, 3, 5, 10, and 30% w/v) on an FR-1 schedule of reinforcement. Testing days occurred every other day and a maintenance session occurred on the intervening non-test days.

### Jugular vein catheterization

Following the completion of assessments measuring goal-directed behavior, jugular vein catheterization was performed using the methods reported by Bertrand et al.^46^. In brief, HIV-1 Tg and control animals were anesthetized using 5% inhalant sevofluorane and maintained at 3.5–4% sevofluorane throughout the surgical procedure. After anesthesia induction, a sterile IV catheter, which extended dorsally and connected to an acrylic pedestal embedded with mesh, was implanted into the right jugular vein and secured with sterile sutures (4–0 Perma–Hand silk; EthiconEnd-Surgery, Inc.). The dorsal portion of the catheter/backmount was implanted subcutaneously above the right and left scapulae and stitched into place using sterile, absorbable sutures (4–0 Monoweb). Immediately following surgery, post-operative analgesia was provided by butorphenol (Dorolex; 0.8 mg/kg, SC; Merck Animal Health, Millsboro, DE) and the antibiotic gentamicin sulfate (0.2 ml 1%, IV; VEDCO, Saint Joseph, MO) was administered to prevent infection. Rats were monitored in a heat-regulated warm chamber following surgery and returned to the colony room after recovery from anesthesia. Two HIV-1 Tg animals died immediately following surgery; one of unknown causes and one suffered from a seizure after returning to the home cage, yielding Control Vehicle, *n* = 10; Control SE, *n* = 11; HIV-1 Tg Vehicle, *n* = 9; HIV-1 Tg SE, *n* = 10 for assessment of drug-seeking behavior.

For one week following surgery, catheters were “flushed” daily with a solution containing heparin (2.5%; APP Pharmaceuticals, Schamburg, IL) and the antibiotic gentamicin sulfate (1%) to prevent clotting and infections, respectively. Seven days after jugular vein catheterization, animals began assessments of drug-seeking behavior and resumed treatment with either SE or vehicle. Prior to operant testing each day, catheters were “flushed” with 0.9% saline solution (Baxter, Deerfield, IL). After the completion of daily operant testing, catheters were “flushed” with post-flush solution.

### Drug-seeking behavior: addiction

#### Response escalation

A hallmark of addiction is the progressive increase in the frequency and amount of drug intake^50^. Two phases (i.e., FR-1 Responding and PR Responding), modified from Morgan et al.^51^ and previously employed in our laboratory by Bertrand et al.^46^, were utilized to produce an escalation of cocaine-maintained responding; an experimental paradigm that affords a critical opportunity to model a key aspect of drug addiction in humans.

First, rats responded for cocaine (0.2 mg/kg/inf) according to a FR-1 schedule of reinforcement for 5 consecutive days (Session Length: 1 h). Second, rats responded for cocaine (0.75 mg/kg/inf) on a PR schedule of reinforcement, whereby the ratio requirement was defined using the exponential function defined above for 14 consecutive days (Maximum Session Length: 120 Minutes). During both FR-1 and PR responding, a 20 s time-out (i.e., active levers were retracted and the house light was extinguished) following the completion of a response requirement.

#### Progressive-ratio responding: dose response

Under a PR schedule of reinforcement, the response requirements are increased immediately following the delivery of a reinforcer^48^ affording a method to evaluate reinforcing efficacy^49^.

Cocaine concentrations (0.01, 0.05, 0.1, 0.2, 0.75, and 1.0 mg/kg/inf) were presented in ascending order. A maintenance session (i.e., 0.2 mg/kg/inf on an FR-1 schedule of reinforcement) occurred every other day.

### Concurrent choice self-administration

After establishing a history of sucrose and cocaine maintained-responding, choice behavior was evaluated using a concurrent choice self-administration experimental paradigm for seven consecutive days. Throughout the experimental procedure, animals were responding for 5% (w/v) sucrose or 0.2 mg/kg/inf of cocaine. On the eighth day, saline was substituted for cocaine. On the ninth day, water was substituted for sucrose. Sucrose-paired lever presentation (right or left) was balanced between groups.

Each session began with four forced-choice trials (i.e., only one “active” lever was available) where animals responded for two sucrose and two cocaine reinforcers. Following the forced choice trials, both “active” levers were concurrently available to allow the animals to freely choose between sucrose and cocaine. After a response was made, a 20 s time-out occurred.

Data for the concurrent choice self-administration were censored due to the absence of responding, and thus absence of a choice, independent of genotype group. Censoring reduced group sample sizes by approximately 50%. Statistical power estimates for a three-way interaction (Genotype × Reinforcer × Day) investigating the impact of the HIV-1 transgene on choice behavior was 0.31; an estimate which is significantly lower than appropriate statistical power levels (i.e., 0.8;^52^) and thus precluded drawing any reliable inferences about choice behavior.

### Neuronal and dendritic spine morphology in medium spiny neurons of the nucleus accumbens

#### Preparation of tissue

Animals were transcardially perfused within 24 h of their last self-administration session. After animals were deeply anesthetized using sevoflurane (Abbot Laboratories, North Chicaco, IL), transcardial perfusion was conducted using the methodology reported by Roscoe et al.^21^ with one minor modification. Specifically, after the brains were dissected, they were post-fixed in 4% paraformaldehyde for 10 min.

#### DiOListic labeling and confocal imaging

MSNs from the NAc were visualized using a ballistic labeling technique, originally described by Seabold et al.^53^. Methodology for the preparation of DiOlistic cartridges, preparation of Tefzel tubing, and DiOlistic labeling was previously described in detail^21^. MSNs were analyzed from the NAc, located approximately 2.28 mm to 0.60 mm anterior to Bregma^54^. Z-stack images were obtained using methodology previously reported^21^.

#### Dendritic branching complexity

Dendritic branching was manually evaluated for images with a clear dendritic arbor, as assessed by the maximum intensity projection image. Analyses were conducted on one to four MSNs per animal, yielding Control Vehicle, *n* = 7; Control SE, *n* = 7; HIV-1 Tg Vehicle, *n* = 5; HIV-1 Tg SE, *n* = 10. Given the nested experimental design, cluster means were calculated for dendritic branching, whereby the sample size (*n*) reflects the number of animals in each group.

#### Dendritic spine morphology quantification

Dendritic spine morphological parameters, including dendritic spine length^19^ and dendritic spine head^17,18^, are strongly correlated with synaptic strength and area of the postsynaptic density, respectively. Assessing how the HIV-1 transgene and/or SE alter dendritic spine morphology following a history of cocaine self-administration, therefore, affords a critical opportunity to evaluate the functional features of spine synapses^20^.

Dendritic spine parameters were analyzed using the AutoNeuron and AutoSpine extension modules in Neurolucida360 (MicroBrightfield, Williston, VT, USA). Spine analyses were conducted for MSNs meeting the following selection criteria, including 1. Continuous staining beginning in the cell body extending throughout the dendrite; 2. Minimal DiI diffusion; and 3. Low background fluorescence. Analyses were conducted on one to eight MSNs per animal, yielding Control Vehicle, *n* = 10; Control SE, *n* = 11; HIV-1 Tg Vehicle, *n* = 6; HIV-1 Tg SE, *n* = 10. Given the nested experimental design, cluster means were calculated for morphological parameters, whereby the sample size (*n*) reflects the number of animals in each group.

Two dendritic spine morphological parameters, including dendritic spine backbone length (µm) and dendritic spine head diameter (µm), were assessed. Dendritic spines were included in the analysis if they met the definitional criteria established using previously published manuscripts (i.e., backbone length, 0.2 to 4.0 µm^55^; head diameter, 0.0 to 1.2 µm^56^; volume, 0.05 to 0.85 µm^3^^57^).

### Statistical analysis

Data were analyzed using analysis of variance (ANOVA) and regression techniques (SAS/STAT Software 9.4, SAS Institute, Inc., Cary, NC; SPSS Statistics 27, IBM Corp., Somer, NY; GraphPad Software, Inc., La Jolla, CA). An alpha criterion of *p* ≤ 0.05 was utilized to establish statistical significance. Orthogonal decompositions and/or the Greenhouse–Geisser df correction factor^58^ were implemented to account for variables that may violate the compound symmetry assumption. Based on the a priori aims of the present study, planned comparisons were conducted to evaluate the impact of chronic HIV-1 viral protein exposure on goal-directed and drug-seeking behavior (i.e., Control Vehicle vs. HIV-1 Tg Vehicle), the presence of an SE effect (i.e., HIV-1 Tg Vehicle vs. HIV-1 Tg SE), and the magnitude of the SE effect (i.e., Control Vehicle vs. HIV-1 Tg SE).

For goal-directed behavior, the a priori planned comparisons were addressed using regression and/or ANOVA techniques. Specifically, regression analyses were conducted to evaluate the shape and parameters of the best-fit function for the acquisition of autoshaping, PR responding, and FR-1 responding. To further evaluate the impact of chronic HIV-1 viral protein exposure on PR responding, a mixed-model ANOVA was conducted using SPSS Statistics 27. Two dependent variables of interest, including the number of reinforcers and the number of “active” lever presses, were investigated. In cases where an animal was tested more than once at a single concentration, a cluster mean was calculated to account for the nested experimental design^59,60^. The mean series imputation method was used for occasionally censored data, which occurred at the 1% (HIV-1 Tg SE: *n* = 1) and 10% (Control Vehicle: *n* = 1) concentration. One HIV-1 Tg animal treated with vehicle failed to acquire autoshaping and therefore did not complete either the PR or FR assessment; the animal was not included in the statistical analyses for these tasks.

For drug-seeking behavior, regression and/or ANOVA techniques were also utilized to statistically evaluate the a priori planned comparisons. FR-1 responding for a 0.2 mg/kg/inf dose of cocaine was evaluated using regression analyses and a mixed-model ANOVA (PROC MIXED; SAS/STAT Software 9.4) with an unstructured covariance structure. The number of infusions earned and number of “active” lever presses during the cocaine PR task (0.75 mg/kg/inf dose) were evaluated using regression analyses. Additionally, a mixed model ANOVA (PROC MIXED; SAS/STAT Software 9.4) with a compound symmetry covariance structure was also conducted to assess the number of “active” lever presses during the cocaine PR task. Regression analyses were used to statistically analyze the number of cocaine infusions during the dose–response cocaine PR assessment.

Neuronal morphology and dendritic spine morphological parameters were analyzed using ANOVA techniques. Specifically, dendritic spine branch order was evaluated using a mixed-model ANOVA (SPSS Statistics 27) and dendritic spine backbone length and head diameter were analyzed using a generalized linear mixed effects model with a Poisson distribution and an unstructured covariance structure (PROC GLIMMIX; SAS/STAT Software 9.4).

## Results

### Presence of the HIV-1 transgene

#### Preliminary training

**Figure 2:**
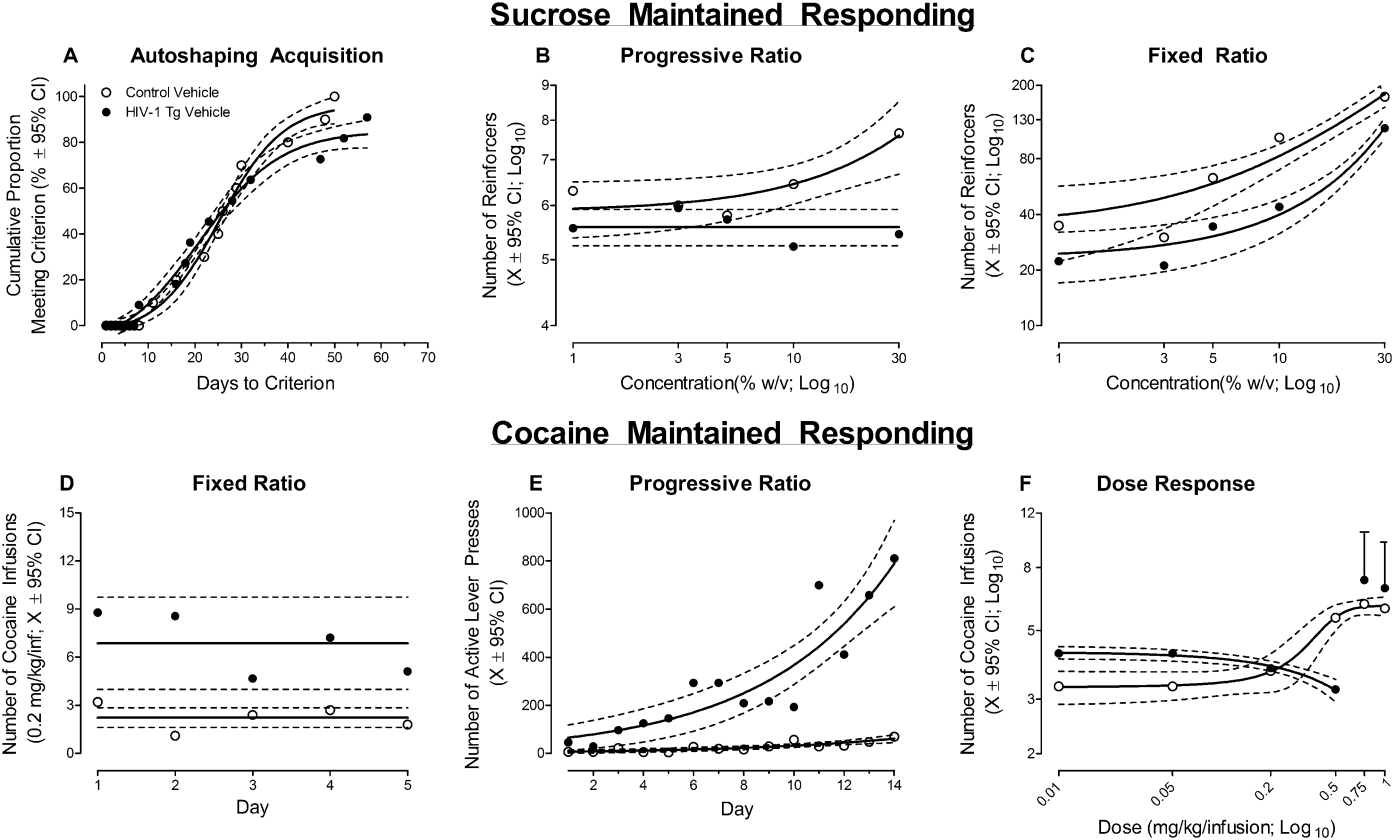
The impact of the HIV-1 transgene on sucrose-maintained responding (**A–C**) and cocaine-maintained responding (**D–F**) is illustrated as a function of genotype (HIV-1 Tg Vehicle vs. Control Vehicle; ± 95% Confidence Intervals). HIV-1 Tg animals took significantly longer to successfully acquire autoshaping, supporting a profound deficit in stimulus-reinforcement learning (**A**). Under sucrose-maintained responding, apathetic behaviors in the HIV-1 Tg rat were characterized by a diminished sensitivity to (**B**), and reinforcing efficacy of (**C**), sucrose relative to control animals. Under cocaine-maintained responding, HIV-1 Tg animals exhibited an increased response vigor, independent of schedule of reinforcement (i.e., Fixed Ratio 1: **D**; Progressive Ratio: **E** and **F**), relative to control animals. Furthermore, an increased sensitivity to cocaine dose was observed in HIV-1 Tg animals relative to controls (**F**). Collectively, independent of differences in the unique response to either sucrose or cocaine self-administration, the HIV-1 Tg rat exhibits prominent alterations in goal-directed behaviors.

*Acquisition of Autoshaping.* At the genotypic level, HIV-1 Tg animals treated with vehicle acquired autoshaping, earning at least 60 reinforcers for three consecutive days, significantly slower than control animals treated with vehicle (Fig. 2A). After 66 days, 100% of the control and 90% of the HIV-1 Tg rats treated with vehicle met criteria. A sigmoidal-dose response curve (variable slope) afforded the best-fit for the number of days to criteria for both HIV-1 Tg (*R*^*2*^ = 0.98) and control (*R*^*2*^ = 0.98) animals treated with vehicle; albeit statistically significant differences in the parameters of the function [*F*(4, 27) = 4.6, *p* ≤ 0.006] were observed. Results support, therefore, a prominent deficit in stimulus-reinforcement learning in HIV-1 Tg rats treated with vehicle.

#### Goal-directed behavior: apathy

##### Progressive-ratio responding: dose-response

After successfully acquiring autoshaping, the reinforcing efficacy of sucrose was evaluated using a dose–response experimental design and PR schedule of reinforcement. HIV-1 Tg animals treated with vehicle exhibited a diminished reinforcing efficacy of sucrose relative to control animals treated with vehicle evidenced by a statistically significant genotype × concentration interaction for both the number of reinforcers (Fig. 2B; [*F*(4, 68) = 3.4, *p*_GG_ ≤ 0.02, η^2^_p_ = 0.165] with a prominent linear component [*F*(1,17) = 6.7, *p* ≤ 0.02, η^2^_p_ = 0.282]) and “active” lever presses (Data Not Shown; [*F*(4, 68) = 2.9, *p*_GG_ ≤ 0.05, η^2^_p_ = 0.147] with a prominent linear component [*F*(1,17) = 6.0, *p* ≤ 0.03, η^2^_p_ = 0.261]. Specifically, control animals treated with vehicle displayed a linear increase in the number of reinforcers (*R*^*2*^ = 0.87) and “active” lever presses (*R*^*2*^ = 0.93) as sucrose concentration increased. The number of reinforcers earned and “active” lever presses produced by HIV-1 Tg animals treated with vehicle, however, remained invariant across sucrose concentration, well-described by a horizontal line. HIV-1 Tg animals, therefore, exhibit a diminished reinforcing efficacy of sucrose.

##### Fixed-ratio responding: dose-response

The sensitivity to sucrose concentration was subsequently examined using an FR-1 schedule of reinforcement. HIV-1 Tg animals treated with vehicle exhibited a blunted sensitivity to sucrose concentration relative to control animals treated with vehicle (Fig. 2C), evidenced by a differential pattern of responding. Regression analyses revealed a dose–response function well-characterized by a one-phase association in control animals treated with vehicle (*R*^*2*^ = 0.95), whereas an exponential growth equation afforded a best-fit for HIV-1 Tg animals treated with vehicle (*R*^*2*^ = 0.98).

Collectively, results support prominent apathetic behaviors towards natural reinforcers in HIV-1 Tg rats; apathetic behaviors that are characterized by a diminished reinforcing efficacy of, and sensitivity to, sucrose.

#### Drug-seeking behavior: addiction

##### Response escalation: fixed-ratio responding

Following jugular vein catheterization, HIV-1 Tg and control animals responded for 0.2 mg/kg/inf of cocaine according to a FR-1 schedule of reinforcement for 5 consecutive days. HIV-1 Tg animals treated with vehicle displayed increased response vigor, independent of day, relative to control animals treated with vehicle (Fig. 2D; Main Effect: Genotype, [*F*(1,17) = 4.5, *p* ≤ 0.05]). Furthermore, the number of cocaine infusions across day was well-described by a horizontal line for both HIV-1 Tg and control animals treated with vehicle; albeit statistically significant differences in the mean of the function were observed [*F*(1,93) = 11.1, *p* ≤ 0.01]). Thus, HIV-1 Tg animals treated with vehicle exhibited increased response vigor for cocaine, supporting enhanced drug-seeking behavior, relative to control rats treated with vehicle.

##### Response escalation: progressive-ratio responding

Independent of genotype, both HIV-1 Tg and control animals treated with vehicle escalated their drug intake, evidenced by an increase in the number of “active” lever presses (Fig. 2E) and the number of cocaine infusions (Data Not Shown; 0.75 mg/kg/inf), across the 14 consecutive days of PR testing.

With regards to the number of “active” lever presses, HIV-1 Tg animals treated with vehicle escalated responding at a significantly faster rate relative to control animals treated with vehicle (Day × Genotype interaction, [*F*(1, 245) = 12.0, *p* ≤ 0.01]). An exponential growth equation afforded the best fit for the number of “active” lever presses for both HIV-1 Tg (*R*^*2*^ = 0.82) and control (*R*^*2*^ = 0.73) animals treated with vehicle; albeit statistically significant differences in the parameters of the function were observed [*F*(2,24) = 66.5, *p* ≤ 0.01].

Collectively, results support enhanced drug-seeking behavior in HIV-1 Tg animals, evidenced by an increased response vigor and faster escalation, relative to control animals treated with vehicle; an observation that is independent of schedule of reinforcement (i.e., FR vs. PR).

##### Progressive-ratio responding: dose-response

HIV-1 Tg animals treated with vehicle exhibited an increased reinforcing efficacy of cocaine dose (mg/kg/inf) relative to control animals treated with vehicle (Fig. 2F). Specifically, in HIV-1 Tg animals treated with vehicle, a linear (*R*^*2*^ = 0.98) decrease in the number of cocaine infusions was observed from the 0.01 to 0.5 mg/kg/inf dose; a dramatic increase in the number of infusions was observed at the 0.75 mg/kg/inf and 1 mg/kg/inf dose. In sharp contrast, in control animals treated with vehicle, the number of cocaine infusions across all doses was well-described by a sigmoidal-dose response curve (variable slope; *R*^*2*^ = 0.99). Collectively, results support enhanced drug-seeking behavior and an increased differential reinforcing efficacy of cocaine in HIV-1 Tg rats treated with vehicle relative to control rats treated with vehicle.

#### Neuronal and dendritic spine morphology in medium spiny neurons of the nucleus accumbens

##### Dendritic branching complexity

**Figure 3:**
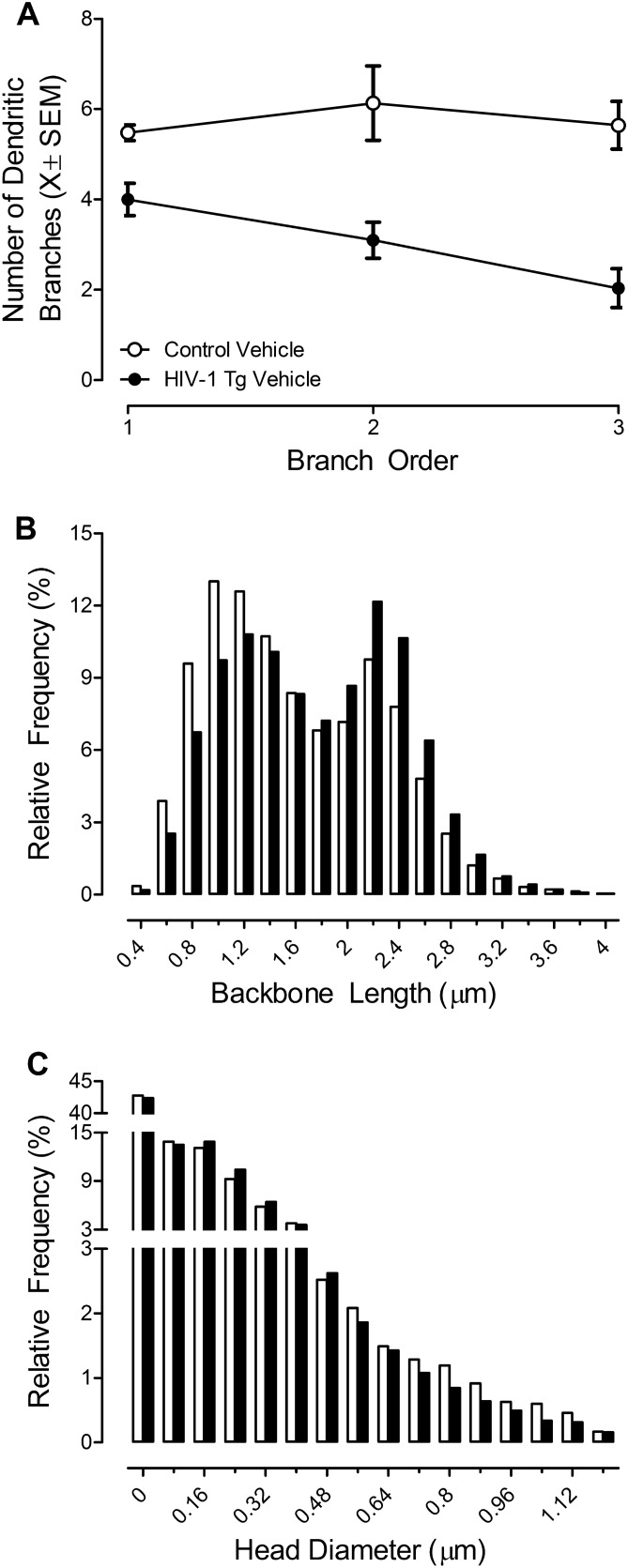
Following a history of cocaine self-administration, the impact of the HIV-1 transgene on dendritic branching (**A**; ± SEM) and dendritic spine morphology (**B–C**) in medium spiny neurons of the nucleus accumbens was examined and is illustrated as a function of genotype (HIV-1 Tg Vehicle vs. Control Vehicle). HIV-1 Tg animals exhibited a dramatic decrease in dendritic branching complexity (**A**) and a population shift towards longer dendritic spines (**B**) with decreased head diameter (**C**) relative to control animals.

Dendritic branching was evaluated by counting the number of primary, secondary, and tertiary branches in MSNs of the NAc. Following chronic HIV-1 viral protein exposure, lower levels of dendritic branching were observed in HIV-1 Tg animals treated with vehicle relative to control animals treated with vehicle (Fig. 3A; Genotype × Branch Order interaction with a prominent linear component, [*F*(1,10) = 7.3, *p* ≤ 0.02, η^2^_p_ = *0.*422]). Thus, presence of the HIV-1 transgene leads to lower levels of dendritic branching in MSNs of the NAc.

##### Dendritic spine morphology

After a history of cocaine self-administration, dendritic spine morphology was examined in MSNs of the NAc. HIV-1 Tg animals treated with vehicle exhibited a prominent population shift towards longer dendritic spines (Fig. 3B; [*F*(1,1274) = 581.6, *p* ≤ 0.01]) with decreased head diameter (Fig. 3C; [*F*(1,1070) = 119.9, *p* ≤ 0.01]) relative to control animals treated with vehicle. Presence of the HIV-1 transgene, therefore, shifts the morphological parameters of dendritic spines in MSNs of the NAc to a more immature phenotype.

### Therapeutic efficacy of S-Equol

#### Preliminary training

##### Acquisition of autoshaping

**Figure 4:**
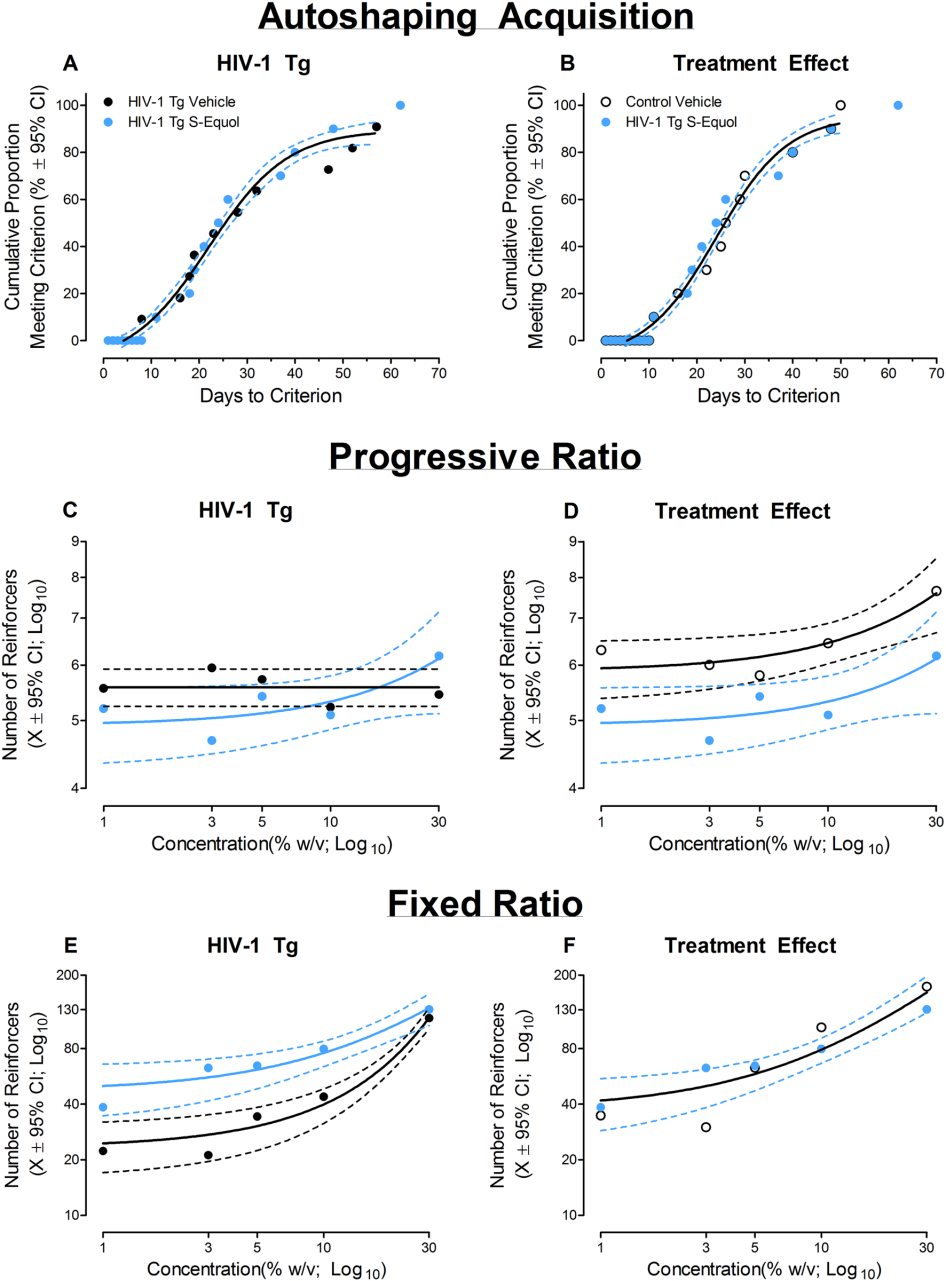
The therapeutic efficacy of S-Equol (SE) as a novel therapeutic for apathetic behaviors under sucrose-maintained responding in the HIV-1 Tg rat is illustrated as a function of genotype (HIV-1 Tg vs. Control) and treatment (SE vs. Vehicle; ± 95% Confidence Intervals). The number of days required to successfully acquire autoshaping, an index of stimulus-reinforcement learning, for HIV-1 Tg animals treated with SE was statistically indistinguishable from either HIV-1 Tg animals treated with vehicle (**A**) or control animals treated with vehicle (**B**). Treatment with SE enhanced the reinforcing efficacy of sucrose (**C–D**) and ameliorated alterations in the sensitivity to sucrose (**E–F**) in HIV-1 Tg rats.

Treatment with SE ameliorated deficits in stimulus-reinforcement learning in HIV-1 Tg animals. All HIV-1 Tg animals treated with SE successfully acquired autoshaping. The best-fit function (i.e., global sigmoidal dose–response curve (variable slope)) for the number of days to criteria for HIV-1 Tg animals treated with SE was statistically indistinguishable from either HIV-1 Tg animals treated with vehicle (Fig. 4A; *p* > 0.05; *R*^*2*^ = 0.98) or control animals treated with vehicle (Fig. 4B; *p* > 0.05; *R*^*2*^ = 0.98). Thus, the marked impairment in stimulus-reinforcement learning observed in HIV-1 Tg animals treated with vehicle was mitigated by treatment with SE.

#### Goal-directed behavior: apathy

##### Progressive-ratio responding: dose response

The reinforcing efficacy of sucrose in HIV-1 Tg animals was enhanced by treatment with SE. First, comparison of HIV-1 Tg animals treated with SE and HIV-1 Tg animals treated with vehicle revealed a differential pattern of responding. Specifically, HIV-1 Tg animals treated with SE exhibited a linear increase in the number of reinforcers (Fig. 4C; *R*^*2*^ = 0.73) and “active” lever presses (Data Not Shown; *R*^*2*^ = 0.91) as sucrose concentration increased; a concentration-dependent effect not observed in HIV-1 Tg animals treated with vehicle (Best Fit: Horizontal Line).

Second, both HIV-1 Tg animals treated with SE and control animals treated with vehicle exhibited a linear increase in the number of reinforcers (Fig. 4D; *R*^*2*^ = 0.73 and 0.87, respectively) and “active” lever presses (Data Not Shown; *R*^*2*^ = 0.93 and 0.91, respectively) as sucrose concentration increased. Although HIV-1 Tg animals treated with SE exhibited decreased response vigor (i.e., Statistically Significant Difference in β_0_; Reinforcers: [*F*(1,6) = 12.4, *p* ≤ 0.01]; “Active” Lever Presses: [*F*(1,6) = 10.8, *p* ≤ 0.02]) relative to control animals treated with vehicle, the rate of increase (i.e., β_1_) across sucrose concentration was statistically indistinguishable between groups (*p* > 0.05). Treatment with SE, therefore, mitigates the diminished reinforcing efficacy of sucrose observed in older HIV-1 Tg animals.

##### Fixed-ratio responding: dose-response

Alterations in the sensitivity to sucrose were ameliorated by treatment with SE in HIV-1 Tg animals. Comparison of HIV-1 Tg animals treated with SE and HIV-1 Tg animals treated with vehicle (Fig. 4E) revealed differential patterns of responding (i.e., First-Order Polynomial (*R*^*2*^ = 0.95) and Exponential Growth Equation (*R*^*2*^ = 0.98), respectively), whereby HIV-1 Tg animals treated with SE exhibited greater sensitivity to varying sucrose concentrations. Furthermore, the number of sucrose reinforcers earned by HIV-1 Tg animals treated with SE and control animals treated with vehicle (Fig. 4F) was well-described by a global one-phase association (*R*^*2*^ = 0.91) supporting no statistically significant differences between groups in sucrose sensitivity. Taken together, treatment with SE mitigated deficits in stimulus-reinforcement learning and apathetic behaviors in older HIV-1 Tg rats by enhancing the reinforcing efficacy of, and sensitivity to, sucrose.

#### Drug-seeking behavior: addiction

##### Response escalation: fixed-ratio responding

HIV-1 Tg animals treated with SE displayed an initial novelty response to cocaine self-administration followed by a rapid decay, to levels statistically indistinguishable from either HIV-1 Tg or control animals treated with vehicle, in the number of cocaine infusions earned across the five day testing period.

**Figure 5:**
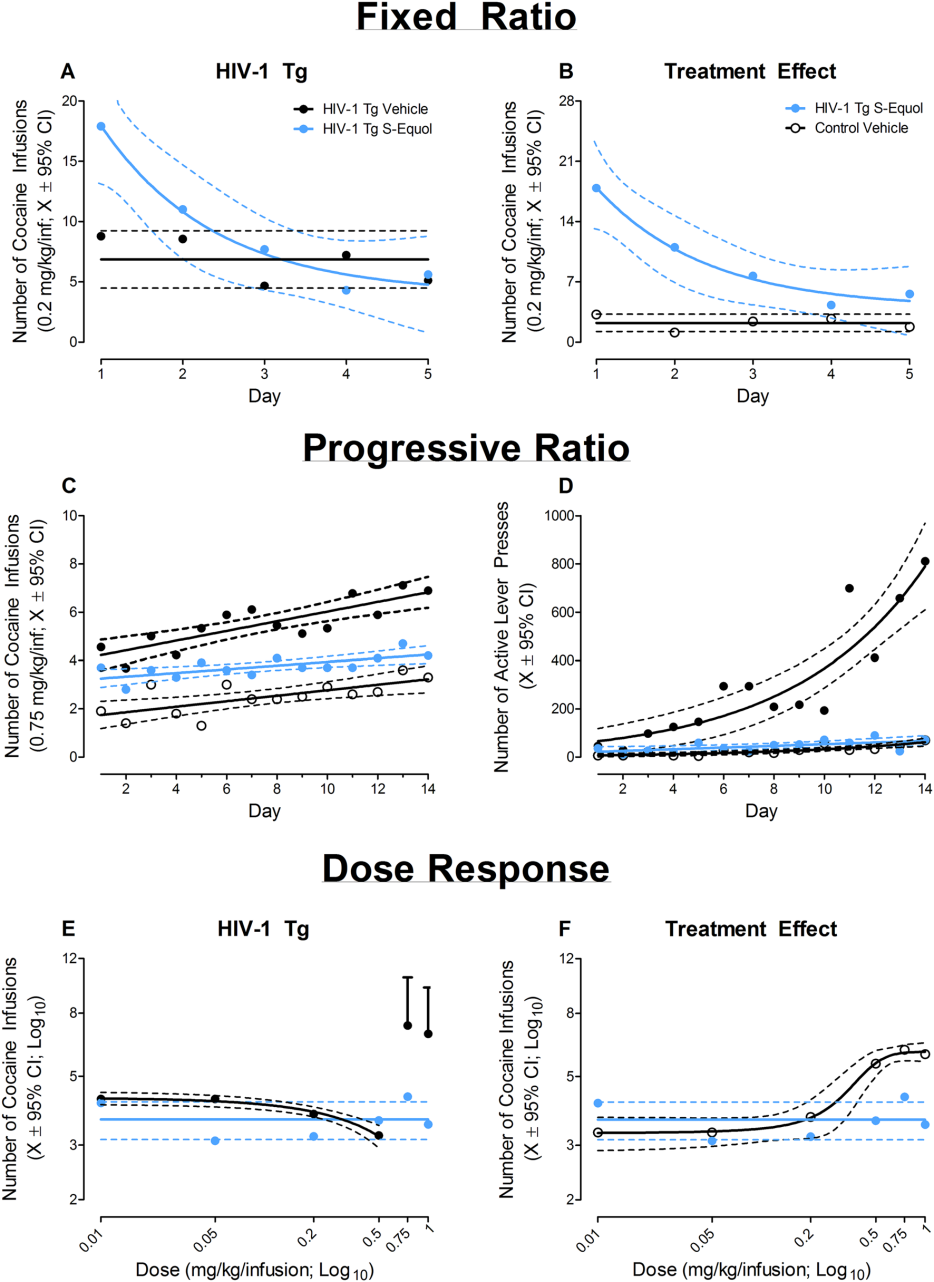
The therapeutic efficacy of S-Equol (SE) as a novel therapeutic for apathetic behaviors under cocaine-maintained responding in the HIV-1 Tg rat is illustrated as a function of genotype (HIV-1 Tg vs. Control) and treatment (SE vs. Vehicle; ± 95% Confidence Intervals). HIV-1 Tg animals treated with SE displayed an initial novelty response to cocaine self-administration followed by a rapid decay. At the end of the five day fixed ratio testing period, the number of cocaine infusions earned by HIV-1 Tg animals treated with SE were statistically indistinguishable from either HIV-1 Tg (**A**) or control animals (**B**) treated with vehicle. Treatment with SE reduced drug-seeking behavior in the HIV-1 Tg rat (**C–D**); an effect which generalized across cocaine dose (**E–F**).

Comparison of HIV-1 Tg animals treated with SE and HIV-1 Tg animals treated with vehicle revealed a differential pattern of responding (Fig. 5A; Treatment × Day interaction, [*F*(1,74) = 4.42, *p* ≤ 0.04]). Specifically, a one-phase decay afforded the best-fit for the number of infusions earned by HIV-1 Tg animals treated with SE (*R*^*2*^ = 0.97), while a horizontal line was most appropriate for HIV-1 Tg animals treated with vehicle. However, the overlapping 95% confidence intervals observed from self-administration days two through five suggest that the differential pattern of responding is driven primarily by an initial novelty response in HIV-1 Tg animals treated with SE on the first day of cocaine self-administration.

A differential pattern of responding (i.e., HIV-1 Tg SE: One-Phase Decay, *R*^*2*^ = 0.97; Control Vehicle: Horizontal Line) was also observed when comparing HIV-1 Tg animals treated with SE and control animals treated with vehicle (Fig. 5B; Genotype × Day interaction, [*F*(1,78) = 12.4, *p* ≤ 0.01]). Again, however, the overlapping 95% confidence intervals observed at self-administration days four and five suggests that the number of cocaine infusions earned by HIV-1 Tg animals treated with SE at the end of FR-1 responding is statistically indistinguishable from control animals treated with vehicle.

##### Response escalation: progressive-ratio responding

Independent of genotype and/or treatment, an escalation of cocaine intake, evidenced by an increase in the number of cocaine infusions (Fig. 5C) and number of “active” lever presses (Fig. 5D), was observed across the 14 consecutive days of PR testing.

Examination of the number of cocaine infusions revealed a linear increase in the number of cocaine infusions for all three groups (Control Vehicle: *R*^*2*^ = 0.48, HIV-1 Tg Vehicle: *R*^*2*^ = 0.69, HIV-1 Tg SE: *R*^*2*^ = 0.49). Treatment with SE, however, mitigated response vigor and drug escalation relative to HIV-1 Tg animals treated with vehicle. Specifically, comparison of HIV-1 Tg animals treated with SE and HIV-1 Tg animals treated with vehicle revealed statistically significant differences in the intercept (i.e., β_0_; [*F*(1, 24) = 5.0, *p* ≤ 0.04]) and in the rate of escalation (i.e., β_1_; [*F*(1,24) = 7.8, *p* ≤ 0.01]) between groups. Albeit, response vigor in HIV-1 Tg animals treated with SE was still significantly greater than control animals treated with vehicle (β_0_: [*F*(1,24) = 30.0, *p* ≤ 0.01]; no statistically significant differences in the rate of escalation (i.e., β_1_) were observed (*p* > 0.05).

With regards to the number of “active” lever presses, HIV-1 Tg animals treated with SE escalated responding significantly slower than HIV-1 Tg animals treated with vehicle (Day × Treatment interaction, [*F*(1,245) = 12.2, *p* ≤ 0.01]). Specifically, HIV-1 Tg animals treated with SE and HIV-1 Tg animals treated with vehicle exhibited differential patterns of escalation, evidenced by different best-fit functions (i.e., First-Order Polynomial, *R*^*2*^ = 0.39 and Exponential Growth Equation, *R*^*2*^ = 0.82, respectively). Critically, the escalation of responding in HIV-1 Tg animals treated with SE was statistically indistinguishable from control animals treated with vehicle (Day × Treatment interaction, *p* > 0.05). Thus, treatment with SE reduced drug-seeking behavior in HIV-1 Tg animals.

##### Progressive-ratio responding: dose-response

Variations in the dose (mg/kg/inf) of cocaine revealed an alteration in the reinforcing efficacy of cocaine in HIV-1 Tg animals treated with SE. Comparison of HIV-1 Tg animals treated with SE and HIV-1 Tg animals treated with vehicle revealed a differential pattern of cocaine self-administration dependent upon dose (Fig. 5E)). Specifically, HIV-1 Tg animals treated with vehicle exhibited prominent dose-dependent changes in responding (i.e., a linear (*R*^*2*^ = 0.98) decrease in the number of cocaine infusions from the 0.01 to 0.5 mg/kg/inf dose followed by a dramatic increase in the number of cocaine infusions at the 0.75 and 1 mg/kg/inf dose); a dose-dependent effect not observed in HIV-1 Tg animals treated with SE (Best Fit: Horizontal Line). A differential pattern of responding (i.e., HIV-1 Tg SE: Horizontal Line; Control Vehicle: Sigmoidal Dose Response (Variable Slope); *R*^*2*^ = 0.99) was also observed when comparing HIV-1 Tg animals treated with SE and control animals treated with vehicle (Fig. 5F). Treatment with SE, therefore, precludes the increase in responding at higher cocaine doses supporting a diminished reinforcing efficacy of cocaine in HIV-1 Tg animals. Furthermore, it is notable that utilization of a dose–response experimental paradigm also supports the generalization of reduced drug-seeking behavior in HIV-1 Tg animals across dose.

#### Neuronal and dendritic spine morphology in medium spiny neurons of the nucleus accumbens

##### Branch order

**Figure 6:**
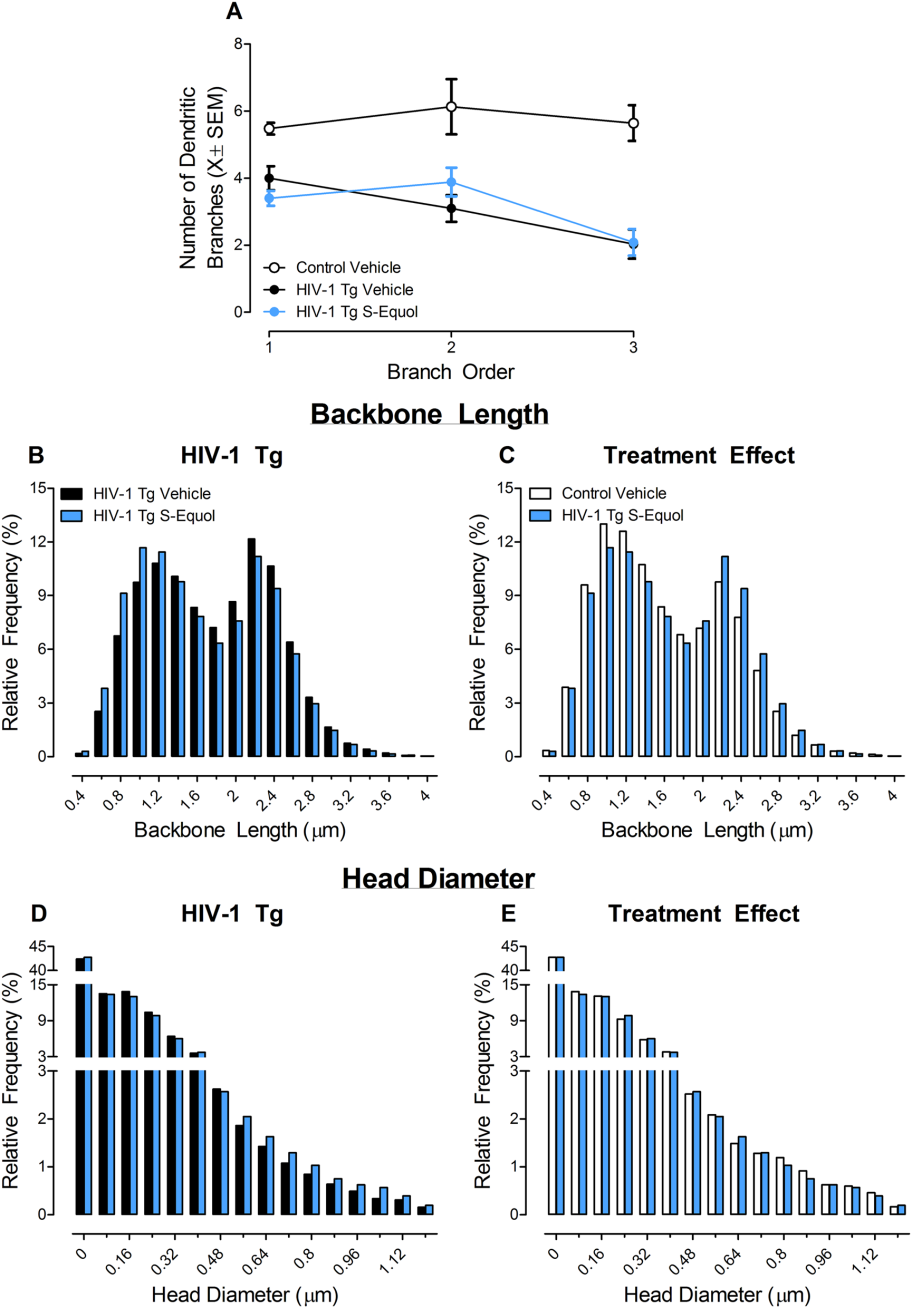
The utility of S-Equol (SE) to modify neuronal (**A**; ± SEM) and dendritic spine morphology (**B–E**) in medium spiny neurons of the nucleus accumbens is illustrated as a function of genotype (HIV-1 Tg vs. Control) and treatment (SE vs. Vehicle; ± 95% Confidence Intervals). Although treatment with SE failed to alter dendritic branching (**A**), long-term modifications in dendritic spine morphology were observed in HIV-1 Tg animals, evidenced by a prominent population shift towards shorter dendritic spines (**B**) with increased head diameter (**D**) relative to HIV-1 Tg animals treated with vehicle. Treatment with SE shifted the morphological parameters of dendritic spines in MSNs of the NAc to a more mature phenotype relative to HIV-1 Tg animals treated with vehicle, evidenced by a prominent population shift towards shorter dendritic spines (Fig. 6B; [*F*(1,1538) = 79.6, *p* ≤ 0.01]) with increased head diameter (Fig. 6D; [*F*(1,1292) = 25.4, *p* ≤ 0.01]). Relative to control animals treated with vehicle, HIV-1 Tg animals treated with SE exhibited significantly longer dendritic spines (**C**), no statistically significant differences in head diameter (**E**) between the two groups were observed.

Treatment with SE failed to increase the level of dendritic branching complexity in HIV-1 Tg animals in MSNs of the NAc following a history of cocaine self-administration (Fig. 6A). Specifically, dendritic branching complexity in HIV-1 Tg animals treated with SE was indistinguishable from HIV-1 Tg animals treated with vehicle (*p* > 0.05) and remained significantly lower relative to control animals treated with vehicle (Genotype × Branch Order interaction with a prominent linear component, [*F*(1,15) = 5.3, *p* ≤ 0.04, η^2^_p_ = *0.*259]).

##### Dendritic spine morphology

Morphological parameters, including backbone length and head diameter, of dendritic spines in medium spiny neurons (MSNs) of the nucleus accumbens (NAc) were enhanced in HIV-1 Tg animals treated with SE. Treatment with SE shifted the morphological parameters of dendritic spines in MSNs of the NAc to a more mature phenotype relative to HIV-1 Tg animals treated with vehicle, evidenced by a prominent population shift towards shorter dendritic spines (Fig. 6B; [*F*(1,1388) = 216.2, *p* ≤ 0.01]) with increased head diameter (Fig. 6D; [*F*(1,1166) = 112.7, *p* ≤ 0.01]). Furthermore, although HIV-1 Tg animals treated with SE exhibited significantly longer dendritic spines (Fig. 6C; [*F*(1,1498) = 155.5, *p* ≤ 0.01]) relative to control animals treated with vehicle, no statistically significant differences in head diameter between the two groups were observed (Fig. 6E; *p* > 0.05). Thus, treatment with SE induced long-term modifications in dendritic spine morphology resulting in a more mature morphological phenotype.

## Discussion

HIV-1 Tg rats treated with vehicle exhibited alterations in goal-directed and drug-seeking behavior relative to their control counterparts, supporting prominent motivational alterations; alterations that were mitigated by treatment with SE. At the genotypic level, apathetic behavior in HIV-1 Tg rats treated with vehicle was characterized by a diminished reinforcing efficacy of, and sensitivity to, sucrose. Motivational alterations in the HIV-1 Tg rat were further evidenced by enhanced drug-seeking for cocaine, supporting an addictive phenotype. Treatment with SE, however, ameliorated alterations in goal-directed and drug-seeking behaviors in HIV-1 Tg rats. The therapeutic benefits of SE may be due, at least in part, to the partial restoration of synaptic function, evidenced by a population shift towards a more mature dendritic spine phenotype in HIV-1 Tg animals treated with SE. Taken together, SE restored motivated behavior in HIV-1 Tg rats, expanding the potential clinical utility of SE to include both neurocognitive and affective alterations.

The assessment of goal-directed^61^ and drug-seeking^62^ behavior, as in the present study, relies upon Pavlovian conditioning and operant, or instrumental, conditioning. Pavlovian conditioning utilizes repeated associations of two stimuli to activate behavior. With reference to operant conditioning, the utilization of either “positive” (i.e., the addition of a stimulus following a response) or “negative” (i.e., the removal of a stimulus following a response) reinforcement increases the likelihood that the response will occur again^63^. Once conditioning processes were learned (e.g., via autoshaping acquisition), an aspect of reward expectation (e.g., unit-dose of the reward; schedule of reinforcement: FR vs. PR) was manipulated to elucidate changes in the motivated behavior resulting from either HIV-1 viral proteins and/or SE treatment.

Constitutive expression of HIV-1 viral proteins induced apathetic and addictive behaviors affording strong evidence for motivational alterations. Apathetic behaviors for a natural reward were evidenced by a diminished reinforcing efficacy of, and sensitivity to, sucrose. Notably, the behavioral presentation of apathy in the present study is distinct from the decreased response vigor previously reported in young (i.e., 2 months of age) HIV-1 Tg rats^46^. Given the positive correlation between apathy and age in HIV-1 seropositive individuals^64^, differences in the behavioral presentation of apathy may be due, at least in part, to age; albeit there were also notable differences in experimental design. Addictive behaviors in HIV-1 Tg rats treated with vehicle were characterized by an enhanced response vigor for cocaine. The enhanced response vigor for cocaine observed in the present study is again distinct from observations by Wayman et al. (^65^; i.e., no statistically significant difference in responding) or Bertrand et al. (^46^); i.e., reduced response vigor); inconsistencies which may reflect differences in age, the experimental protocol, and/or cocaine dose. However, the observed increased sensitivity to cocaine in older HIV-1 Tg rats is consistent with previous observations in relatively young (i.e., 3–4 months of age) HIV-1 Tg rats^66^. Therefore, the differences in sensitivity to cocaine between the Bertrand et al.^46^ manuscript and the present study more likely results from differences in cocaine dose. Collectively, independent of differences in the response to either sucrose or cocaine self-administration across studies, the HIV-1 Tg rat exhibits prominent motivational alterations supporting an advantageous biological system for the evaluation of therapeutics for apathy resulting from chronic HIV-1 viral protein exposure.

Motivational alterations resulting from chronic HIV-1 viral protein exposure were mitigated by treatment with SE; results which extend the therapeutic utility of SE. The therapeutic efficacy of SE, a selective ERβ agonist, for neurocognitive impairments associated with HAND has been critically tested across multiple ages (i.e., treatment beginning at PD 28, 2–3 months of age, and 6–8 months of age), neurocognitive domains (e.g., temporal processing, stimulus-reinforcement learning, sustained attention), and the factor of biological sex^32–34^. Results of the present study extend the therapeutic utility of SE to include the mitigation of apathetic behaviors, as evidenced by the enhanced reinforcing efficacy of, and sensitivity to, sucrose observed in HIV-1 Tg rats treated with SE. Furthermore, treatment with SE dramatically reduced drug-seeking behavior in the HIV-1 Tg rat; a finding that deserves further consideration given the well-recognized relationship between estrogens and drug abuse^67^.

Women are uniquely vulnerable to cocaine addiction, evidenced by a faster acquisition of cocaine self-administration^68,69^, greater willingness-to-work for cocaine (i.e., breakpoint;^70^) and a greater sensitivity to cocaine^71,72^; preclinical observations which are consistent with the clinical picture of addiction^73^. The enhanced behavioral responses to cocaine result, at least in part, from the activation of ERβ in the NAc^74^. Notably, when control animals are treated with SE, an agonist selective for ERβ^26^, an increased reinforcing efficacy of, and altered sensitivity to, cocaine is observed (Supplementary Fig. 1A–C). The structure and function of the NAc, however, is altered by chronic HIV-1 viral protein exposure [e.g.,^11,22,75,76^], which may preclude this untoward side effect (i.e., enhanced drug-seeking behavior) in HIV-1 Tg rats treated with SE.

Examination of dendritic spine morphology in MSNs of the NAc following a history of cocaine self-administration affords additional evidence for structural alterations in the NAc in the HIV-1 Tg rat. Dendritic spines, tiny specialized protrusions that emerge from dendritic shafts, are the primary postsynaptic target for excitatory synaptic transmission^77^. Morphologically, dendritic spines are classically divided into four primary categories, including thin, stubby, mushroom and filopodia^78^; albeit the classification system has significant limitations given that, in reality, spine morphology occurs along a continuum^20^. Strong correlations between dendritic spine morphology (e.g., head volume, neck length) and area of the post-synaptic density (PSD)^16–18^, presynaptic vesicles^16,17^, as well as synaptic strength^19^ support a tight coupling between morphological parameters and function. Assessments of dendritic spine morphology, therefore, may provide critical information on synaptic function^20^.

Following a history of cocaine self-administration, HIV-1 Tg animals treated with vehicle exhibited a population shift towards longer dendritic spines with decreased dendritic spine head diameter relative to control animals treated with vehicle; a population shift consistent with a ‘filopodia’-like morphology. Unstable, immature filopodia often lack a clear dendritic spine head and asymmetric synapse implying that these spines also lack synaptic contacts^79^. However, treatment with SE led to long-term modifications in dendritic spines in MSNs of the NAc in HIV-1 Tg animals. Specifically, HIV-1 Tg animals treated with SE exhibited a prominent population shift towards a more mature phenotype (i.e., ‘thin’) relative to HIV-1 Tg animals treated with vehicle. Flexible thin spines have a small synapse, but have structural flexibility to accommodate changes in inputs^80^. Given that motivation is mediated in part by the NAc^13,81^, the shift in dendritic spine morphology affords a potential mechanism by which SE exerts its therapeutic effects. Critically, the beneficial effects of SE on the morphological parameters of dendritic spines in the HIV-1 Tg rat may reflect the utility of SE to more broadly remodel neuronal circuitry.

Indeed, the ovarian steroid hormone 17β-estradiol acts on multiple brain regions (i.e., PFC, NAc) associated with the fronto-striatal circuit. In the PFC, 17β-estradiol increases dendritic spine density [e.g.,^82,83^] via the ERβ pathway^83^ and induces morphological alterations in dendritic spines^82^. Within the NAc, however, 17β-estradiol significantly reduces dendritic spine density in the NAc core and increases the prevalence of immature spine types (i.e., stubby, filopodia;^29,30^). In a consistent manner, in the present study, control animals treated with SE exhibited a population shift towards a less mature dendritic spine phenotype relative to control animals treated with vehicle (Supplementary Fig. 1D–F). To more comprehensively evaluate if and/or how SE remodels neuronal circuitry in the HIV-1 Tg rat, studies in the absence of cocaine and across additional brain regions involved in the fronto-striatal circuit are needed.

Assessments in the present study were limited to female OVX animals, a notable caveat that affords key opportunities for future research. First, prominent sex differences in cocaine addiction^73^ and HAND^84^ are well-recognized. To date, how biological sex influences HIV-1 associated motivational alterations remains understudied supporting the need for additional research. Second, there are notable sex differences in the expression levels of ER-β in the brain^85^. However, no statistically significant differences in the expression levels of ER-β have been observed in brain regions associated with the fronto-striatal circuit [PFC:^85^; Striatum:^86^]. Finally, there remains a critical need to evaluate the therapeutic efficacy of SE for motivational alterations in male and intact female HIV-1 Tg animals. Although the phytoestrogen SE exhibits strong affinity for ERβ^26,27^, it also binds 5α-dihydrotestosterone^87^ supporting its potential efficacy in both males and females. Indeed, with regards to HAND, SE serves as an efficacious neuroprotective therapeutic in both male and intact female animals^33^.

An additional caveat that merits further discussion is the utilization of sucrose as an exemplar of a natural reinforcer. The sweet taste test, which utilizes the random presentation of multiple sucrose concentrations^88,89^ or sweet stimuli^90^ to evaluate hedonic responses and sensitivity to sucrose, is commonly employed to assess anhedonia in clinical populations. Although anhedonia is a prominent diagnostic criterion of multiple neuropsychiatric disorders [e.g., depression^91^], hedonic responses for sucrose in these populations are not statistically different from those observed in healthy controls^88–90^. Therefore, sucrose may not be the most translationally relevant reinforcer for the evaluation of anhedonia. The assessment of goal-directed behavior in the present study, however, requires a motivational component (i.e., lever-pressing for reward) that is not necessary for the sweet taste test. Nevertheless, there remains a critical need to more comprehensively evaluate apathy following chronic HIV-1 viral protein exposure via novel reinforcers (e.g., voluntary wheel running).

In conclusion, following chronic HIV-1 viral protein exposure, prominent motivational deficits and dysregulation, as well as a population shift towards an immature dendritic spine phenotype were observed. In HIV-1 Tg animals, treatment with SE mitigated motivational alterations. Furthermore, morphological parameters of dendritic spines in MSNs of the NAc in HIV-1 Tg animals treated with SE were shifted towards a more mature phenotype, supporting a potential underlying basis for the therapeutic effects of SE for motivational alterations. Taken together, SE restored motivated behavior in the HIV-1 Tg rat, expanding the potential clinical utility of SE to include both neurocognitive and affective alterations.

## Supplementary Information

Supplementary Figure.

## Data Availability

All relevant data are within the paper.
