# Supplementary material for: S-Equol mitigates motivational deficits and dysregulation associated with HIV-1
Source: Sci Rep. 2021 Jun 4;11:11870. doi: 10.1038/s41598-021-91240-0 (PMC8178385; doi:10.1038/s41598-021-91240-0)
Supplement: Supplementary file 1 — Supplementary Figure. [file 41598_2021_91240_MOESM1_ESM.pdf]

S-Equol Mitigates Motivational Deficits and Dysregulation Associated with HIV-1

Kristen A. McLaurin, Sarah J. Bertrand, Jessica M. Illenberger, Steven B. Harrod,
Charles F. Mactutus, Rosemarie M. Booze

Program in Behavioral Neuroscience
Department of Psychology
Barnwell College
1512 Pendleton Street
University of South Carolina
Columbia, SC 29208

Number of Tables, Figures: 0, 6

Running Head (≤48 characters): Motivational Alterations Associated with HIV-1

Submitting and Corresponding Author:
Rosemarie M. Booze, Ph.D.
Professor and Bicentennial Endowed Chair of Behavioral Neuroscience
Department of Psychology
1512 Pendleton Street
University of South Carolina
Columbia, SC 29208
E-mail: booze@mailbox.sc.edu

SUPPLEMENTARY FIGURE 1

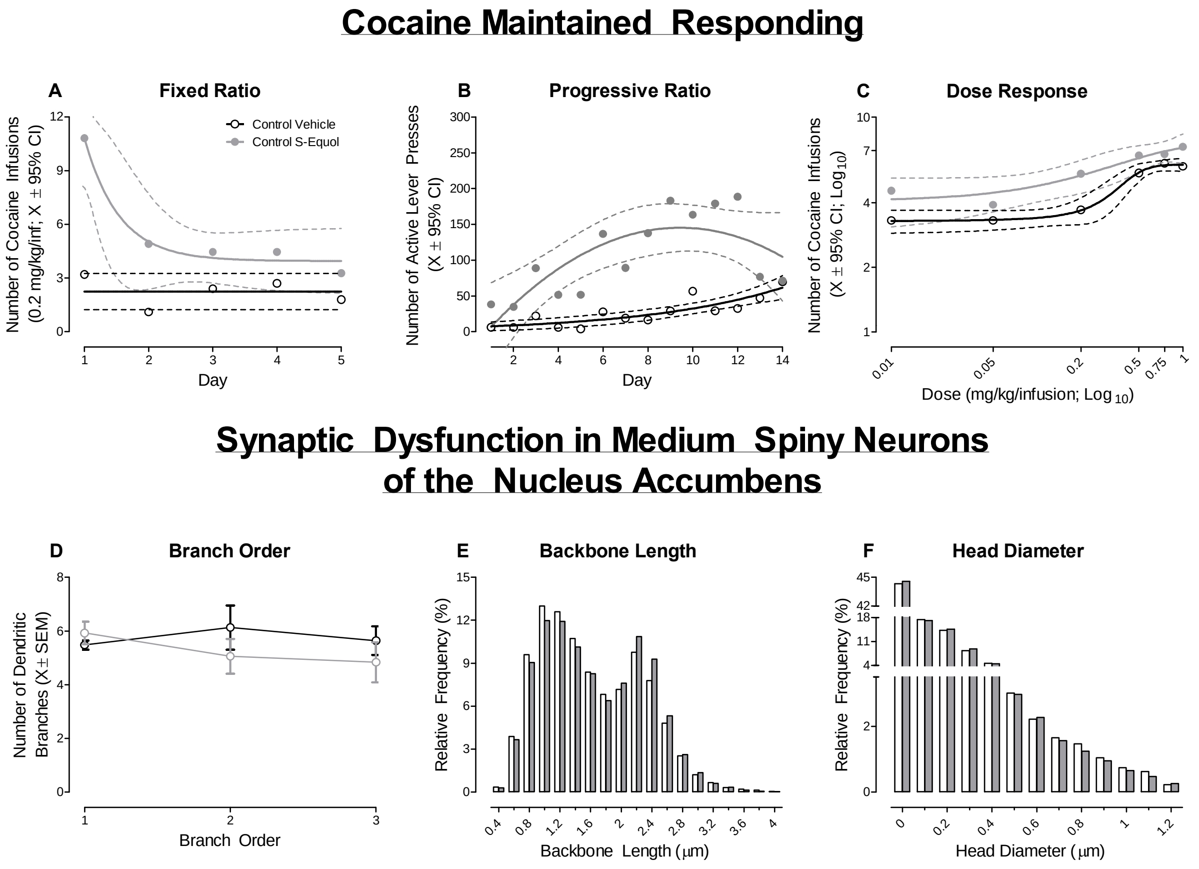

Supplementary Figure S1. The impact of S-Equol (SE) on drug-seeking behavior (**A-C**) and medium spiny neurons (MSNs) of the nucleus accumbens (**D-F**) is illustrated for control animals as a function of treatment (Control Vehicle vs. Control SE). Control animals treated with SE exhibited an initial novelty response to cocaine self-administration followed by a rapid decay (**A**). Treatment with SE increased drug-seeking behavior in control animals (**B**) and altered the sensitivity to cocaine (**C**). Furthermore, although SE had no significant effect on dendritic branching in MSNs (**D**), profound population shifts towards longer dendritic spines (**E**) with decreased head diameter (**F**) were observed relative to control animals treated with vehicle.
